# Accuracy and Validity of 3D Markerless Motion Capture Compared to Marker-Based Systems for Lower-Limb Biomechanical Assessment: A Systematic Review

**DOI:** 10.3390/s26123956

**Published:** 2026-06-22

**Authors:** Aditya Chougule, Matthew Dowsett, David Ekundayomi, Ava Machesney, Tomos Mather, Benjamin Gompels, Stephen McDonnell

**Affiliations:** Trauma and Orthopaedics Department, University of Cambridge, Cambridge CB21TN, UK; mjmd4@cam.ac.uk (M.D.); doe21@cam.ac.uk (D.E.); ava.machesney@nhs.net (A.M.); tllm2@cam.ac.uk (T.M.); bdg30@cam.ac.uk (B.G.); sm2089@cam.ac.uk (S.M.)

**Keywords:** ACL injury, markerless motion capture, biomechanics, injury screening, lower-limb kinematics

## Abstract

Marker-based motion capture systems are considered the gold standard for biomechanical analysis of movements associated with anterior cruciate ligament (ACL) injury risk; however, their cost and technical requirements limit their use for large-scale athlete screening. Markerless motion capture has emerged as a potential alternative, using pose estimation algorithms or depth cameras to quantify movement without reflective markers. This systematic review evaluated the accuracy and validity of markerless motion capture systems for measuring lower-limb kinematics during jump-landing tasks commonly used in ACL injury screening. MEDLINE, Embase, and Web of Science were searched from 1990 to March 2025 for studies comparing markerless and marker-based systems in healthy participants. Extracted outcomes included Bland-Altman plots, root mean square error, mean absolute error, Pearson’s correlation coefficient, coefficient of multiple correlation, and intraclass correlation coefficient. Across studies, markerless systems demonstrated moderate to high validity for several lower-limb kinematic measures, particularly in the sagittal plane, although validity varied across joints, movement phases, and task complexity. These findings suggest markerless motion capture shows potential for biomechanical assessment in ACL injury screening, but further validation is required before widespread implementation.

## 1. Introduction

ACL injury is one of the most serious and prevalent knee injuries in sport [1]. Mechanically, ACL injury occurs when the applied load exceeds the structural tolerance of the ligament. This may happen acutely during a single high-magnitude loading event or cumulatively through repetitive cycles of heavy loading that produce fatigue-associated failure in the absence of recovery [2]. Cutting movements and single-leg landings are the most common activities associated with non-contact ACL rupture, often preceded by rapid deceleration [3]. Approximately 90% of ACL injuries occur through non-contact or indirect contact mechanisms, demonstrating that most ACL injuries arise through excessive intrinsic biomechanical loading patterns without external collision forces [4]. Therefore, preseason screening for ACL injury risk has been proposed as a strategy to guide targeted preventative interventions. Cost-effectiveness modelling across multiple sports suggests that neuromuscular training of all athletes could reduce the incidence of ACL injury from approximately 3% to 1.1% per season [5]. However, despite proven efficacy under controlled conditions, real-world adoption and compliance with injury prevention programs remains low, partly due to time demands and interference with sport-specific training [6]. Consequently, screening approaches that identify high-risk athletes may improve implementation of targeted prevention strategies. Supporting this approach, Myer et al. demonstrated that female athletes classified as high-risk based on elevated knee abduction moment (KAM) during a drop vertical jump (DVJ) derived greater prophylactic benefit from neuromuscular training than their low-risk counterparts [7].

The current gold standard for determining ACL injury risk is laboratory-based three-dimensional motion analysis using marker-based motion capture and force plates. This approach enables precise calculation of joint kinematics linked to ACL loading [8]. In his landmark prospective study, Hewett et al. demonstrated that elevated KAM measured during a DVJ predicted future ACL injury in female athletes, establishing laboratory 3D analysis as a powerful tool for injury risk identification [9]. However, marker-based systems are resource intensive, requiring expensive equipment, skilled personnel, and significant processing time [10]. This limits accessibility to elite or research settings. Additionally, laboratory tasks may not fully replicate the demands of in-game scenarios. Video analyses of basketball injuries have demonstrated that situational factors and player behavior are difficult to reproduce under controlled testing conditions [11]. Markerless motion capture has emerged as a novel alternative to marker-based systems, where pose-estimation algorithms are used to reconstruct 3D joint coordinates from camera footage without the use of reflective markers [12]. Alternatively, depth cameras such as Kinect can be used, which provide depth maps directly [13]. Markerless approaches eliminate time-consuming setup and improve ecological validity by minimizing interference with natural movement due to markers [14]. The reduced environmental constraints for data collection enable analysis of larger cohorts at a lower cost, as well as the potential assessment of athletes in training or competitive environments [15]. This systematic review will evaluate the accuracy and validity of markerless motion capture technologies against the gold standard to provide conclusions on whether markerless performance is interchangeable with marker-based for the purpose of screening for ACL injury risk.

## 2. Methods

**Figure 1:**
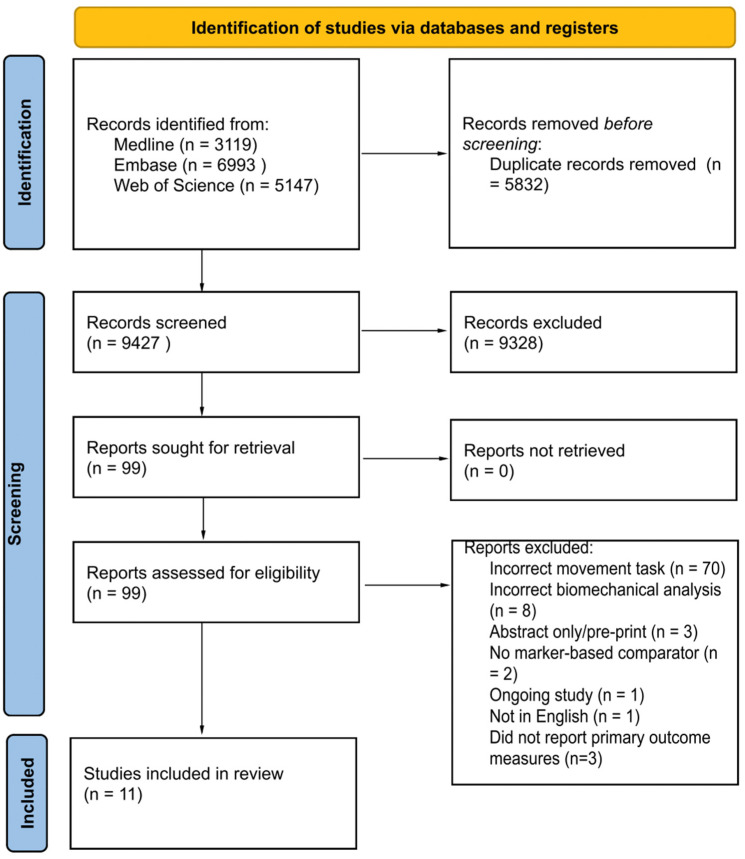
PRISMA flow diagram illustrating the study selection process for the systematic review.

This systematic review was conducted in accordance with the Preferred Reporting Items for Systematic Reviews and Meta-Analyses (PRISMA) guidelines, and the protocol was registered with PROSPERO (CRD420251011431). Figure 1 establishes the PRISMA flow diagram with the protocol observed in selecting studies for this review. On 18 March 2025, a literature search was performed across MEDLINE, Embase, and Web of Science, covering studies published between 1990 and March 2025. Each database was searched individually rather than through a multi-database interface, and search strategies were tailored to account for differences in controlled vocabulary and indexing terms. Searches were limited to studies involving human participants and published in the English language. Retrieved records were imported into Rayyan software for deduplication.

Studies were considered eligible if they compared marker-based motion capture systems with markerless motion capture technologies in healthy individuals of any age. Studies involving clinical populations, including individuals with neuromuscular disorders or those undergoing rehabilitation, were excluded. Randomized controlled trials, cohort studies, case-control studies, and case series were included, while systematic reviews, cadaveric studies, and animal studies were excluded. To maintain relevance to ACL injury risk screening, only studies assessing single-leg or double-leg jumping and landing, and change of direction tasks were included, and papers that evaluated other lower-limb movements, such as gait analysis, were excluded. Additionally, only studies reporting kinematic outcomes were included. Kinetic variables were excluded due to the requirement of specialized equipment such as force plates, which are less suitable for screening applications due to portability demands.

Title and abstract screening were conducted independently by two reviewers (AC and DE), with disagreements resolved by a third reviewer (MD). Articles meeting the initial eligibility criteria underwent full-text review by three independent reviewers (AC, DE, and MD). Data extraction was performed independently by two reviewers (DE and MD) using standardized tables in Microsoft Excel. Extracted variables included movement tasks, biomechanical variables, motion capture methods, participant demographics, and outcome measures relating to accuracy and validity. Summary tables were constructed to capture key metrics. Due to heterogeneity across study designs and methodologies, comparisons were synthesized narratively, with conclusions drawn where two or more studies reported comparable outcome measures.

The methodological quality of each included study was independently appraised by two reviewers (AC, MD) using the COSMIN Risk of Bias tool for studies on measurement error (version 1.0, December 2020), with disagreements resolved by consensus. Each study was evaluated against eight standards encompassing design requirements (S1–S6) and statistical methods (S7–S8), with each standard rated as very good, adequate, inadequate, or not applicable; an overall quality rating was then assigned using the worst-score-counts principle, whereby the lowest rating across applicable standards determined the final classification.

The outcome measures used in this study are summarized below.

Root Mean Square Error (RMSE) quantifies the average magnitude of error between measurements obtained from the markerless system and the marker-based reference. RMSE is calculated as the square root of the mean of the squared differences between corresponding measurements. While no universally accepted standard exists for clinically acceptable RMSE values, it has been suggested that RMSE ≤ 5° is acceptable for lower limb kinematic evaluation as this falls within the typical error range of marker-based systems [16]. RMSE values between 5–10° may be acceptable for gross movement screening but indicate reduced precision, while values > 10° are typically viewed as outside clinically tolerable limits for joint-angle assessment [17].

Mean Absolute Error (MAE) quantified the average absolute difference between measurements from markerless and marker-based systems, providing an estimate of overall numerical deviation without considering direction of error. Similarly to RMSE, MAE ≤ 5° may be considered acceptable for kinematic evaluation as the literature suggests this approximates the inherent inaccuracy of marker-based tools [18]. Errors between 5–10° may still be tolerated in screening contexts, whereas errors exceeding ~10° are frequently regarded as too large for reliable clinical interpretation [18].

Bland-Altman analysis was used to assess agreement between two measurement methods by plotting the mean of paired measurements against their difference. This allows identification of systematic bias and the limits of agreement, representing the range within which 95% of differences between methods are expected to lie. For lower limb joint angles, small biases (<±2–5°) and narrow LoA (on the order of a few degrees) are generally regarded as acceptable for clinical or applied biomechanical use [19]. Wider LoA (>±10°) suggest that the markerless system’s individual measurements may not be reliably interchangeable with those of the reference system [19].

Intraclass Correlation Coefficient—absolute agreement [ICC(2,1)] was used to assess the extent to which measurements obtained from the markerless system were in absolute agreement with those from the marker-based reference. This form of ICC evaluates how closely different measurement methods assign the same values to the same subjects, with higher values indicating stronger agreement between systems. ICC (2,1) values for validity are interpreted in the following manner: poor (<0.5), moderate (0.5–0.75), good (0.75–0.9), and excellent agreement (>0.9) [20].

Pearson’s Correlation Coefficient (PCC) was used to evaluate the strength and direction of the linear relationship between joint-angle waveforms derived from markerless and marker-based systems. Associated *p*-values were used to determine statistical significance. Values closer to 1 indicate a strong positive correlation, meaning the markerless system closely replicates the temporal shape and pattern of the movement measured by the marker-based system [21]. Values near 0 indicate little to no linear relationship between the two waveforms, suggesting poor agreement in how the movement pattern is represented [21].

Coefficient of Multiple Correlation (CMC) was used to assess similarity between kinematic waveforms by quantifying how closely the trajectories obtained from markerless systems matched those from the marker-based reference across time. A markerless system with high CMC values will have strong agreement in the shape and timing of joint-angle trajectories with the marker-based reference [22].

## 3. Results

### 3.1. Study and Population Characteristics

Upon completion of full-text screening, 11 studies were determined to fit the inclusion criteria. The earliest study included was from 2013, and five of the 11 studies were published in 2024. Across all 11 studies, there were a total of 298 participants, with nine studies having between 11 and 20 participants and two studies having greater than 20 participants. Of note, Asaeda et al. and Templin et al. had single-sex cohorts [23,24]. Across the studies, the majority of cohorts recruited had an average age between 20 and 30 years. The most popular movement task analyzed by the included studies is the drop vertical jump (DVJ), followed by the counter movement jump (CMJ). The remaining studies looked at variations of jumping and landing maneuvers. All studies other than Tipton et al. and Turner et al. focused on only one movement [25,26]. Table 1 below provides a summary of the study characteristics. Table 2 demonstrates the COSMIN risk of bias assessment.

### 3.2. Characteristics of Motion Capture Technologies

Vicon and Qualisys were the marker-based motion capture technologies used in the studies, chosen by eight studies and three studies, respectively. Microsoft Kinect V2 was explored by three studies and operates with an infrared time-of-flight depth sensor for three-dimensional tracking. The original Microsoft Kinect was used by two studies and differs in that this sensor utilizes a pattern of actively emitted infrared light and an infrared-sensitive camera to generate a depth image. Other systems used include MMPose, MediaPipe Pose, Sbsq-pose, DeepLabCut, ENABLE, and OpenCap. These technologies use a deep learning approach to pose recognition. Alongside these, the hardware used for motion capture included iPhones and cameras from Sony, Qualisys, and Vicon.

### 3.3. Accuracy

Accuracy in the context of our question reflects how precisely a markerless system can replicate kinematic data derived from a marker-based system [34]. The accuracy of markerless systems was determined by either root mean square error (RMSE) or mean absolute error (MAE). RMSE was the most frequently seen, with four studies opting for this, while MAE was used in three studies.

#### 3.3.1. Root Mean Square Error

Table 3 demonstrates the RMSE values across four studies that compared markerless and marker-based biomechanical data during jump-landing tasks. Aleksic et al., Drazan et al., and Templin et al. used Qualisys as the marker-based reference system, while Turner et al. used Vicon [24,31,33]. Aleksic et al. evaluated MMPose during a countermovement jump and reported RMSE values of 5.4–6.49° for ankle angles, 6.6–7.3° for knee angles, and 8.0–8.8° for hip angles during the entire movement, with higher errors consistently observed on the right side. Drazan et al. assessed DeepLabCut during a vertical jump, reporting RMSE values of 1.96° for hip, 2.68° for knee, and 3.22° for ankle flexion/extension. Templin et al. examined the Enable system during a drop vertical jump and reported RMSE values ranging from 2.5° for hip adduction/abduction to 9.61° for ankle adduction/abduction. Turner et al. evaluated OpenCap across three hopping and landing tasks, measuring hip, knee, and ankle angles in the coronal and sagittal planes of movement [26]. During the double-leg jump-landing rebound task, RMSE ranged from 2.39° for hip adduction/abduction to 6.43° for ankle flexion/extension. The single-leg forward hop produced the smallest RMSE of 3.52° for hip adduction/abduction and the largest of 5.94° for ankle flexion/extension. The last task was a single-leg lateral-vertical hop where the RMSE ranged from 5.02° for hip flexion/extension to 6.87° for ankle flexion/extension.

#### 3.3.2. Mean Absolute Error

Table 4 demonstrates the MAE values across three studies that compared markerless and marker-based biomechanical data during jump-landing tasks. All studies reporting MAE used Vicon as the reference marker-based system. Barzyk et al. paired Vicon with Sbsq-pose during a countermovement jump and reported MAE values of 2.6° for hip, 2.1° for knee, and 4.5° for ankle flexion/extension [32]. Eltoukhy et al. evaluated Kinect V2 during a side-cut maneuver and reported MAE values below 2° for all hip and knee angles measured during both the full movement and the early deceleration phase [30]. Turner et al. compared OpenCap with Vicon across three hopping and landing tasks, reporting hip, knee and ankle angles in the coronal and sagittal planes of movement. During the double-leg jump-landing rebound task, the MAE values ranged from 1.91° for hip adduction/abduction to 5.03° for ankle flexion/extension. The single-leg forward hop produced MAE values ranging from 2.75° for hip adduction/abduction to 4.32° for ankle flexion/extension. The single-leg lateral-vertical hop had a smallest MAE of 3.75° for hip flexion/extension and largest of 5.41° for ankle flexion/extension.

### 3.4. Validity

Validity in this review refers to how well a markerless system represents human movement compared with a marker-based reference [34]. This includes both numerical accuracy and concurrent validity, the ability to capture the same underlying biomechanical pattern [34]. Metrics such as Bland-Altman plots, Intraclass Correlation Coefficient—absolute agreement [ICC(2,1)], Pearson Correlation Coefficient (PCC), and Coefficient of Multiple Correlation (CMC) reflect this pattern-level agreement and therefore indicate validity beyond raw angle accuracy.

#### 3.4.1. Bland-Altman Plots

Table 5 demonstrates the Bland-Altman Plot values across three studies that compared markerless and marker-based biomechanical data during jump-landing tasks. Bland-Altman analysis was used to assess systematic bias, representing the average offset between systems, along with limits of agreement (LoA), which indicate the range within which 95% of individual differences lie. All studies compared different combinations of motion capture techniques and involved different movement tasks. Aleksic et al. compared Qualisys with MMPose during the countermovement jump and reported the smallest bias for ankle angles (−0.49° and −0.67° for the left and right ankles, respectively) and the largest bias for hip angles (3.9° and 5.6° for the left and right hips). Knee-angle bias was 2.6° and 2.01° for the left and right knees. The LoA for the left ankle was −8.6° to 7.7°, and for the right, this was −9.5° to 8.14°. Knee angles showed LoA of −6.7° to 11.9° (left) and −7.3° to 11.31° (right), while hip angles ranged from −6.1° to 14.02° (left) and −3.9° to 15° (right). Asaeda et al. compared Vicon with MediaPipe Pose during single-leg jump-landing and reported a bias of −19.28° for absolute knee valgus angle, with limits of agreement ranging from −12.91° to 25.66° [23]. For knee valgus extrusion from initial contact, the bias was 0.18°, with limits of agreement of 3.17° to 3.53°. Using Vicon and Kinect, Tipton et al. evaluated knee flexion and knee valgus across three tasks: single-leg drop, double-leg drop with 90° pivot, and single-leg hops [25]. Reported biases for knee flexion ranged from 7.39° to 10.4° for single-leg hops and single-leg drop, respectively. For knee valgus, bias ranged from 4.22° for single-leg hops to 5.31° for single-leg drop. For single-leg drop, knee flexion LoA was −11.7° to 26.8° and knee valgus LoA was −8.0° to 13.9°. The double-leg drop with 90° pivot produced knee flexion LoA of −17.7° to 21.3° and knee valgus LoA of −6.3° to 12.6°. The LoA for knee flexion during single-leg hops was −17.8° to 19.7°, and for knee valgus this was −5.9° to 11.6°.

#### 3.4.2. Intraclass Correlation Coefficient—Absolute Agreement [ICC(2,1)]

Table 6 demonstrates the ICC values across six studies that compared markerless and marker-based biomechanical data during jump-landing tasks. The most common model used was ICC(2,1), which represents a two-way random effects model evaluating absolute agreement for single measurements. All studies used Kinect or Kinect V2 as their markerless and Vicon as their marker-based technology. Eloukhy et al. assessed the reliability of Kinect V2 during a side-cut maneuver. All joint-angle measurements exceeded 0.75 except hip abduction/adduction (0.643). Gray et al. evaluated Kinect V2 using a DVJ and recorded knee-ankle separation ratio (KASR) at initial contact and peak flexion, reporting values of 0.84 and 0.95, respectively [29]. Mauntel et al. also used a DVJ and examined hip and knee angles at initial contact (IC), during landing, and joint displacement from IC to landing [28]. At initial contact, the highest values were for knee flexion (left: 0.96; right: 0.95) and lowest were for knee valgus (left: −0.19; right: 0.21). During the landing phase, the values ranged from 0.55 for hip adduction to 0.92 for knee valgus. Stone et al. captured a drop vertical jump and measured knee valgus/varus at initial contact and peak knee flexion, as well as KASR at peak flexion, all returning values of approximately 0.89 [27]. Tipton et al. evaluated Kinect across three movement tasks, reporting the highest values during the double-leg drop with 90° pivot (0.759 for knee flexion; 0.717 for knee valgus) and the lowest during the single-leg hop (0.594 for knee flexion; 0.553 for knee valgus). The single-leg drop produced values of 0.654 and 0.589 for knee flexion and knee valgus, respectively.

#### 3.4.3. Pearson Correlation Coefficient (PCC)

Table 7 demonstrates the PCC values across five studies that compared markerless and marker-based biomechanical data during jump-landing tasks. PCC quantifies the strength and direction of the linear relationship between joint-angle waveforms from markerless and marker-based systems, reflecting similarity in overall movement pattern rather than agreement in absolute angle values [21]. Aleksic et al. and Templin et al. compared MMPose and Enable, respectively, against Qualisys and measured ankle, knee, and hip angles. Aleksic et al. analyzed the countermovement jump, reporting *r* values greater than 0.98 across all joints. During the drop vertical jump, Templin et al. reported the lowest *r* values for ankle adduction/abduction (*r* = 0.06) and knee adduction/abduction (*r* = 0.45), whereas the highest *r* values were seen in flexion/extension across all three joints (*r* ≥ 0.97). Asaeda et al. compared MediaPipe Pose with Vicon for a single-leg jump-landing task. Analysis demonstrated *r* values ranging from 0.008 to 0.590 for absolute knee valgus throughout the movement, and *r* values between 0.554 and 0.757 for knee valgus extrusion from IC. Barzyk et al. compared Sbsq-pose with Vicon during countermovement jump and reported *r* values greater than 0.85 for hip, knee, and ankle flexion/extension across the movement. Eltoukhy et al. evaluated Kinect V2 against Vicon during a side-cut maneuver and reported *r* values greater than 0.7 for all joint dimensions measured except hip abduction/adduction during early deceleration (*r* = 0.471); however, this result was deemed not to be statistically significant (*p* > 0.05). Table 7 below demonstrates the results in greater detail.

#### 3.4.4. Coefficient of Multiple Correlation (CMC)

Table 8 demonstrates the CMC values across two studies that compared markerless and marker-based biomechanical data during jump-landing tasks. CMC evaluates how similar the joint-angle time series (waveform) is between markerless and marker-based systems across the full movement cycle, reflecting dynamic validity rather than solely static agreement [22]. Drazan et al. and Turner et al. used CMC to evaluate DeepLabCut and OpenCap, respectively. Drazan et al., using a vertical jump task with Qualisys as the reference, reported CMC values above 0.99 for hip, knee, and ankle angles across the movement. Turner et al. compared OpenCap with Vicon across three movement tasks and demonstrated that hip, knee, and ankle flexion/extension produced CMC values above 0.8 across all movements. Ankle adduction/abduction values ranged from 0.47 to 0.54 across the three tasks, while hip adduction/abduction ranged from 0.65 to 0.78. Hip internal/external rotation had the lowest value of 0.51 during the single-leg forward hop and the highest value of 0.60 during the double-leg jump-landing rebound task.

## 4. Discussion

The aim of this systematic review was to evaluate the accuracy and validity of markerless motion capture technologies against the gold standard marker-based motion capture. Upon completion of full-text screening, 11 studies were determined to fit the inclusion criteria. A variety of key outcome measures were used to evaluate the accuracy and validity of markerless motion capture. Each metric provides insight into different aspects of performance, such as numerical agreement and waveform similarity, relative to marker-based systems. By examining results across these measures, we aim to clarify the extent to which markerless technologies demonstrate acceptable levels of accuracy and validity for biomechanical assessment relevant to ACL injury mechanisms.

### 4.1. Accuracy

#### 4.1.1. Root Mean Square Error

When comparing across papers, study designs varied widely, reflecting differences in markerless algorithms and experimental protocols. This variability limits direct quantitative comparison between studies. Several studies reported RMSE values that were similar in magnitude to the intrinsic error of marker-based systems (Drazan et al., Turner et al., Templin et al.) [24,26,31]. Aleksic et al. found the smallest RMSE for ankle angles and the largest for hip angles when comparing MMPose to Qualisys; the right lower limb joints also consistently demonstrated higher RMSE than the left [33]. Templin et al. observed the largest RMSE for ankle adduction/abduction (9.61° +/− 4.72°) and hip flexion/extension (6.93° +/− 0.27°), while all other joint-plane combinations remain below 6°, demonstrating good agreement between Enable and Qualisys. Drazan et al. reported RMSE values below 3.3° across hip, knee, and ankle joints using DeepLabCut, with ankle flexion/extension producing the highest result. RMSE values of this magnitude suggest the error introduced by the markerless system is small enough to be considered comparable to the intrinsic uncertainty of Qualisys, rather than reflecting true inaccuracy of DeepLabCut. Turner et al. reported consistent RMSE values across all movements and joint planes, with no significant discrepancies highlighted. The largest RMSE observed for each task was in ankle flexion/extension, reaching up to 6.87° +/− 2.00° for single-leg lateral-vertical hop; however, the large uncertainty associated with each result negates indication of poor accuracy associated with OpenCap. All results remain within the magnitude of inherent inaccuracy documented for marker-based systems themselves, arising from soft-tissue artefact and inter-protocol differences in segment and joint-center definitions [35]. Consequently, OpenCap demonstrates consistent agreement with Vicon across the three tasks and various joint dimensions. Furthermore, the complex, multi-planar movements analyzed by Turner et al. highlight the caliber of OpenCap in producing precise joint-angle quantification under dynamic conditions.

#### 4.1.2. Mean Absolute Error

Across the three studies, MAE values were generally low, suggesting close numerical agreement between markerless systems and Vicon. Barzyk et al. studied only sagittal plane kinematics with Sbsq-pose and produced MAE values < 5° across all joints—within the range acceptable for clinical biomechanical analysis [32]. The marginally higher MAE at the ankle compared with the hip and knee may reflect greater sensitivity of distal segments to joint-center estimation errors in markerless pipelines, a trend also observed in RMSE-based analysis (Drazan et al., Templin et al.). The greater sensitivity could be attributed to the small size and anatomical complexity of the ankle joint, as it involves multiple articulations, meaning that small absolute errors in marker placement or segment orientation can translate into proportionally larger angular errors compared with larger joints such as the hip. Additionally, ankle kinematics often exhibit rapid changes in angle and velocity during tasks such as jumping, landing, and cutting, so even minor temporal or spatial inaccuracies in joint-center estimation can be amplified in the resulting joint-angle calculations [36]. Eltoukhy et al. demonstrated particularly small MAE values (<2°) for hip and knee joint-planes with Kinect V2, suggesting very close agreement in average joint-angle magnitudes for the side-cut maneuver [30]. Such low MAE values fall within the range of inherent inaccuracy documented for marker-based systems themselves, supporting the interpretation that observed differences may reflect between-protocol variability driven by soft-tissue artefact and joint-center modelling conventions, rather than systematic error in the markerless system [35]. Turner et al. reported consistently low MAE values (<6°) across the three movement tasks. Similarly to RMSE analysis, ankle flexion/extension MAE values were highest within each movement category, potentially due to the anatomical and biomechanical characteristics of the ankle joint discussed previously. Moreover, soft tissue artefacts are accentuated in the foot and ankle due to thin and mobile soft tissue layers, which may emphasize inaccuracies in joint-center estimation [37]. Overall, the low MAE values reported across these studies provide converging evidence that several markerless systems can reproduce joint-angle magnitudes that closely approximate those obtained from Vicon for common athletic tasks.

### 4.2. Validity

#### 4.2.1. Bland-Altman Plots

Across the three studies employing this measure, substantial variability was observed in both bias magnitude and limits of agreement, highlighting task- and joint-dependent differences in markerless system performance. In Aleksic et al., Bland-Altman plots revealed relatively small mean biases across ankle, knee, and hip joints during the countermovement jump but wide LoA, particularly for hip and knee angles, indicating large variability in individual measurements across the movement cycle. In this case, low bias does not necessarily equate to good agreement of MMPose with Qualisys, as individual differences may still be meaningful. Asaeda et al. reported a very large negative bias for absolute knee valgus angle when comparing MediaPipe Pose to Vicon during single-leg jump-landing; this demonstrates significant underestimation of knee valgus angle by MediaPipe Pose [23]. The extremely wide LoA for absolute knee valgus shows substantial intra-system variability at the individual level. However, it must be noted that the bias recorded lies outside the given LoA, which is not statistically coherent, as the distribution of differences should be centered around the mean bias. Upon inspection, the authors have incorrectly stated the limits of agreement in their study. The Bland-Altman plots in Asaeda et al. demonstrate LoA of −25.66° to −12.91° for absolute knee valgus and −3.165° to 3.526° for knee valgus extrusion. The associated biases are validated by the altered LoA; hence, the discussion points generated from the results are defensible. Tipton et al. further illustrated task-dependent variability in Bland-Altman outcomes when comparing Kinect to Vicon across three landing and hopping tasks [25]. Bias values differed by both movement and joint angle, with knee flexion showing larger bias and wider LoA than knee valgus. This pattern was consistent across tasks, indicating that sagittal plane kinematics involving large ranges of motion may be more prone to markerless estimation error than coronal plane measures, although both exhibited considerable individual-level variability. Furthermore, all bias values across tasks were positive, indicating group-level overestimation of joint angles relative to Vicon, with knee flexion during single-leg drop showing the greatest overestimation; this finding is supported by the positive upper LoA being of greater magnitude than the negative lower LoA across all movement tasks and joint angles, suggesting individual-level bias towards overestimation. Collectively, from our Bland-Altman analysis across studies, we observe substantial variation in agreement by joint, plane of motion, and task, even within the same markerless system. Additionally, the LoA frequently exceeded ±5°, suggesting that while markerless systems may approximate group-level trends, their use for precise individual-level joint-angle assessment remains limited in many contexts.

#### 4.2.2. Intraclass Correlation Coefficient—Absolute Agreement [ICC(2,1)]

Eltouhky et al. reported high values (>0.75) for most hip and knee joint angles during a side-cut maneuver using Kinect V2. Hip abduction/adduction during the early deceleration phase proved an exception with moderate agreement (ICC = 0.643). Moreover, Gray et al. reported high values for KASR at both initial contact (0.84) and peak flexion (0.95) during a drop vertical jump, illustrating excellent agreement between Vicon and Kinect V2 for this composite coronal plane measure [29]. In contrast, Mauntel et al. observed more variable values when examining hip and knee joint angles during a drop vertical jump, with knee flexion being the only measure to consistently exceed 0.75 across both initial contact and landing phases [28]. Other hip and knee measures demonstrated lesser validity, particularly at initial contact. In particular, three of the values are negative, indicating that the measurement error between Kinect V2 and Vicon exceeded the true between-subject variance for these variables. This reflects not simply poor agreement, but a situation where two systems are more dissimilar within individuals than individuals are from one another, rendering the markerless estimate clinically meaningless for those specific outputs. This pattern is most severe for coronal-plane kinematics. The *p*-values associated with these negative ICCs are all highly non-significant, confirming that agreement does not differ from chance and that no reliable signal is being captured. A further inconsistency is the marked asymmetry in ICC between limbs for the same variable: left knee valgus during joint displacement yields −1.52, while the right yields 0.8 (*p* = 0.001). This discrepancy may reflect positional artefact relative to sensor placement but is left unexplained by the authors. Stone et al. demonstrated high values (~0.89) for knee valgus/varus angles and KASR at initial contact and peak knee flexion during a drop vertical jump using Kinect, indicating excellent agreement with Vicon for these coronal plane knee measures [27]. However, Tipton et al. highlighted notable task dependency in validity outcomes, with the single-leg tasks producing lower values than the double-leg movement. This pattern suggests that more complex or unilateral tasks may introduce greater variability in markerless measurements, reducing the validity of Kinect. Collectively, ICC findings across studies indicate that markerless systems such as Kinect V2 can demonstrate good to excellent validity for certain lower-limb kinematic variables, particularly sagittal plane measures. However, validity is not uniform across all joints, planes, and movement tasks, with reduced values observed for hip coronal plane motion, early movement phases, and complex unilateral tasks.

#### 4.2.3. Pearson Correlation Coefficient

PCC was used by multiple studies as a marker of conceptual validity, reflecting whether markerless systems reproduce the same pattern of joint kinematics observed with marker-based motion capture [21]. Across studies, PCC values were generally highest for sagittal plane flexion/extension movements and lower for coronal plane kinematics. Analyzing MMPose, Aleksic et al. reported very high PCC values (>0.98) for hip, knee, and ankle angles across the countermovement jump, indicating that the temporal profile of joint motion demonstrated excellent agreement with Qualisys. However, when considered alongside Bland-Altman and RMSE findings, these results illustrate a limitation of PCC in that high correlation can coexist with systematic bias and absolute error. Hence, PCC alone is insufficient to infer validity, and this underscores the importance of interpreting PCC in conjunction with other metrics. Templin et al. noted high *r* values (>0.97) for flexion/extension movements of the hip, knee, and ankle joints, whereas coronal and transverse plane movements demonstrated markedly lower correlation, in particular, knee abduction/adduction (*r* = 0.45) and ankle adduction/abduction (*r* = 0.06). Similar plane-dependent trends were observed in Asaeda et al. and Barzyk et al. Asaeda et al. analyzed only knee valgus with MediaPipe Pose and reported *r* values below 0.6 throughout the single-leg jump-landing, whereas Barzyk et al. used Sbsq-pose to study only flexion/extension angles across a countermovement jump and reported *r* values greater than 0.85 across hip, knee, and ankle joints. This reinforces the assertion that markerless systems tend to capture the overall pattern of large-amplitude sagittal plane movements more consistently than smaller-magnitude coronal plane motion. Eltoukhy et al. demonstrated that Kinect V2 does not align with the aforementioned pattern. High *r* values (>0.7) were reported across all hip and knee joint movements during the side-cut maneuver. An anomaly is seen in hip adduction/abduction (*r* = 0.471); however, the *p*-value associated with this is greater than 0.05, hence this is not a statistically significant result. Overall, these findings indicate that PCC-derived concurrent validity in markerless motion capture is task- and plane-dependent and must be interpreted alongside measures of absolute agreement to provide a complete assessment of system performance.

#### 4.2.4. Coefficient of Multiple Correlation

Unlike discrete-point measures, CMC reflects how closely the timing, shape, and coordination of kinematic trajectories align between systems and so is used to evaluate dynamic validity [22]. However, CMC does not give an indication of systematic bias or the magnitude of absolute error so cannot provide a complete interpretation of the validity of the markerless system. In Drazan et al., DeepLabCut demonstrated very high CMC values (>0.99) for hip, knee, and ankle flexion/extension during a vertical jump. This indicates near-identical waveform trajectories between DeepLabCut and Qualisys. Importantly, these findings align with the low RMSE values reported in the same study, providing converging evidence that high CMC values in this context were accompanied by small absolute errors. In contrast, Turner et al. reported a wider range of CMC values when evaluating OpenCap against Vicon across three hopping and landing tasks. While hip, knee, and ankle flexion/extension consistently yielded CMC values above 0.8, substantially lower CMC values were observed for coronal and transverse plane movements. When considered across studies, CMC results reinforce a recurring theme in markerless motion capture validation: markerless systems tend to reproduce flexion/extension kinematics with greater accuracy than coronal or transverse plane rotations. However, as with PCC, high CMC values do not preclude the presence of systematic bias or significant absolute error; therefore, they must be interpreted alongside metrics such as RMSE and Bland-Altman analysis to fully characterize validity.

### 4.3. Comparative Performance Across Technologies

Across studies, several markerless motion-capture systems demonstrated accuracy and validity results that were comparable to the uncertainty range of marker-based references. It was noted that performance varied by technology, joint, plane of motion, and movement task. Accuracy metrics indicated that DeepLabCut and OpenCap most consistently produced joint-angle errors within clinically acceptable thresholds (generally ≤5° and rarely exceeding ~7°). This finding was supported by high CMC values, which confirmed that DeepLabCut and OpenCap reproduce the temporal profile of jumping and landing tasks well, particularly for sagittal plane kinematics. Furthermore, Enable and MMPose demonstrated good accuracy with low-to-moderate RMSE values, albeit with greater joint- or plane-specific deviations, making them more appropriate for applied or screening contexts. Both systems produced high PCC values for flexion/extension kinematics, indicating strong concurrent validity in this plane, with Enable also demonstrating moderate agreement in the coronal and transverse planes. Moreover, Kinect V2 exhibited good accuracy and validity. The main findings were small MAE values during a side-cut maneuver (Eltoukhy et al.), supported by high *r* values with PCC analysis, and strong absolute agreement with ICC(2,1), particularly in the sagittal plane. In contrast, MediaPipe Pose exhibited substantial systematic error and variability for knee valgus, suggesting limited accuracy for coronal-plane assessment. Collectively, the evidence suggests that markerless motion capture is currently most accurate and valid for sagittal-plane lower-limb kinematics during both unilateral and bilateral athletic tasks, particularly when using deep learning-based systems such as DeepLabCut and OpenCap. Across technologies, coronal and transverse-plane joint angles, in particular knee valgus, and complex unilateral movements proved the least precise applications, limiting immediate interchangeability with marker-based motion capture.

## 5. Future Work and Limitations

Several limitations should be considered when interpreting the findings of this systematic review. First, there was substantial methodological heterogeneity across studies, with variation in movement tasks, participant characteristics, camera configurations, and biomechanical modelling approaches. The authors acknowledge that when comparing values across studies using different movement tasks, for example, in the countermovement jump in Aleksic et al. and the vertical jump in Drazan et al., differences in reported RMSE cannot be unambiguously attributed to the markerless technology alone, as task-specific kinematic demands will independently influence the magnitude of observed error [31,33]. Accordingly, cross-study comparisons in this review are interpreted with this caveat in mind. Rather than aggregating values across studies to derive a single performance estimate for each technology, the discussion focuses on the direction and relative magnitude of findings within each study, and cross-study findings are framed as indicative trends rather than direct equivalences. Where studies report the same task and technology, comparisons are made with greater confidence; where tasks differ, commentary is restricted to where the order of magnitude of error is broadly consistent across the literature. Furthermore, sample sizes were typically small and predominantly composed of healthy young adults, limiting statistical power and reducing generalizability to pediatric, elderly, or injured populations in whom biomechanical assessment is often relevant. Many studies focused on sagittal and coronal-plane kinematics, while less evidence was available for transverse-plane motion. Together, these factors restrict the conclusions that can be drawn regarding markerless motion capture performance. Future research should provide greater emphasis on ecological validity, as many current validation studies occur in controlled laboratory environments. Markerless motion capture should be evaluated under real-world conditions, including outdoor settings, variable lighting, and occlusion. Direct comparative studies between different markerless technologies are also needed to clarify relative performance and cost-effectiveness. In addition to injury-risk assessment, further longitudinal and clinically oriented investigations would be beneficial in determining whether markerless motion capture can meaningfully inform rehabilitation monitoring and return-to-sport decision-making. Such work is critical for translating the promising technical validity seen in this review into genuine clinical and applied impact.

## 6. Conclusions

Overall, evidence from this review indicates that several markerless motion capture systems can approximate marker-based measurements with acceptable accuracy and validity for selected lower-limb joint angles and movement tasks. Agreement is strongest for sagittal-plane kinematics during jump-landing or hopping activities, where absolute errors are often comparable to the intrinsic uncertainty of marker-based systems and waveform similarity is high. In contrast, reduced validity is observed for coronal and transverse plane motion and more complex or unilateral tasks, with knee valgus elucidating substantial systematic error and variability. Notably, more distal joints offered lower accuracy in all planes. Collectively, these findings suggest that markerless motion capture is approaching practical utility for movement screening in controlled settings, yet broader clinical testing and real-world evaluation are required before it can be confidently adopted for precise biomechanical assessment in athletes.

## Figures and Tables

**Table 1:** Summary of study characteristics.

Authors	Number of Participants			Age (Mean ± *SD*)	Motion Capture Technology			Movement Tasks
	All	Male	Female		Marker-Based	Markerless	Markerless Hardware	
Turner et al. (2024) [26]	12	6	6	18.2 ± 3.3	Vicon	OpenCap	2 iPhone 12 SE	Double-leg jump-landing rebound task, single-leg forward hop, single-leg lateral-vertical hop
Tipton et al. (2019) [25]	20	14	6	30.8 ± 5.9	Vicon	Kinect	1 Kinect sensor	Single-leg drop, double-leg drop with 90° pivot, single-leg hops
Templin et al. (2024) [24]	127	0	127	15.56 ± 1.38	Qualisys	ENABLE	12 Qualisys Mi-qus cameras	Drop vertical jump
Stone et al. (2013) [27]	13	10	3	20–31	Vicon	Kinect	1 Kinect sensor	Drop vertical CMJ
Mauntel et al. (2021) [28]	20	10	10	20.5 ± 2.78	Vicon	Kinect V2	1 Kinect V2 sensor	Drop vertical jump
Gray et al. (2017) [29]	38	20	18	24.79 ± 2.86	Vicon	Kinect V2	1 Kinect V2 sensor	Drop vertical jump
Eltoukhy et al. (2019) [30]	15	6	9	16 ± 0.9	Vicon	Kinect V2	1 Kinect V2 sensor	Side-cut maneuvers
Drazan et al. (2021) [31]	15	Data not given	Data not given	Data not given	Qualisys	DeepLabCut	2 Sony ICX285 cameras	Vertical jump
Barzyk et al. (2024) [32]	11	10	1	28.4 ± 9.2	Vicon	Sbsq-pose	1 iPhone X	Countermovement jump
Asaeda et al. (2024) [23]	15	15	0	19.9 ± 1.0	Vicon	MediaPipe Pose	1 Vicon Vue	Single-leg jump-landing
Aleksic et al. (2024) [33]	12	7	5	25.6 ± 3.4	Qualisys	MMPose	2 iPhone 13	Countermovement jump

**Table 2:** COSMIN Risk of Bias Assessment for Included Studies—Standards for Measurement Error.

Authors	Design Requirements						Statistical Methods		Overall Rating
	S1	S2	S3	S4	S5	S6	S7	S8	
	Patient Stability	Time Interval	Measurement Conditions	Blinding: Administration	Blinding: Scoring	Other Flaws	Continuous Stat. Method	Ordinal Stat. Method	
Aleksic et al. (2024)	NA	NA	VG	NA	A	A	VG	NA	Adequate
Asaeda et al. (2024)	NA	NA	VG	NA	A	I *	A	NA	Inadequate
Barzyk et al. (2024)	NA	NA	VG	NA	A	A	A	NA	Adequate
Drazan et al. (2021)	NA	NA	VG	NA	A	A	A	NA	Adequate
Eltoukhy et al. (2019)	NA	NA	VG	NA	A	A	A	NA	Adequate
Gray et al. (2017)	NA	NA	VG	NA	A	VG	I †	NA	Inadequate
Mauntel et al. (2021)	NA	NA	VG	NA	A	A	I †	NA	Inadequate
Stone et al. (2013)	NA	NA	VG	NA	A	A	I †	NA	Inadequate
Templin et al. (2024)	NA	NA	VG	NA	A	VG	A	NA	Adequate
Tipton et al. (2019)	NA	NA	VG	NA	A	A	VG	NA	Adequate
Turner et al. (2024)	NA	NA	VG	NA	A	A	A	NA	Adequate

Key: VG = Very Good; A = Adequate; I = Inadequate; NA = Not Applicable. Standards: S1: Were patients stable in the time between the repeated measurements on the construct to be measured? S2: Was the time interval between the repeated measurements appropriate? S3: Were the measurement conditions similar for the repeated measurements, except for the condition being evaluated as a source of variation? S4: Did the professional(s) administer the measurement without knowledge of scores from other repeated measurements? S5: Did the professional(s) assign scores or determine values without knowledge of scores from other repeated measurements? S6: Were there any other important flaws in the design or statistical methods of the study? S7: For continuous scores—was the Standard Error of Measurement (SEM), Smallest Detectable Change (SDC), Limits of Agreement (LoA), or Coefficient of Variation (CV) calculated? S8: For ordinal/dichotomous scores—was percentage specific agreement calculated? Notes: S1, S2, S4—Rated Not Applicable for all studies: both measurement systems captured data simultaneously within the same trial, eliminating any between-measurement time interval and rendering sequential administration blinding inapplicable. S5—Rated Adequate for all studies: automated processing pipelines for marker-based and markerless systems operate independently, providing inherent separation of score assignment; no study reported cross-referencing of outputs between systems during scoring. S8—Rated Not Applicable for all studies: all kinematic outcomes are continuous. S7 †—Rated Inadequate for Gray et al., Mauntel et al., and Stone et al., which report ICC alone. ICC(2,1) absolute agreement is a recognized concurrent validity metric in biomechanics and is not inherently inappropriate in method comparison studies; however, it does not satisfy the COSMIN measurement error criterion for S7, which requires an absolute metric expressed in the unit of measurement (degrees). The Inadequate rating reflects this reporting gap (specifically the absence of RMSE, MAE, LoA, or equivalent) rather than a fundamental flaw in study design. Future studies should supplement ICC with at least one absolute error metric to enable meaningful clinical interpretation of measurement precision. RMSE and MAE (reported by Aleksic, Barzyk, Drazan, Eltoukhy, Templin, and Turner et al.) are treated as analogous to SEM and rated Adequate. Bland-Altman limits of agreement (LoA; Aleksic, Asaeda, and Tipton et al.) satisfy the Very Good criterion when the formula is described and the results are coherent. S6 *—Rated Inadequate for Asaeda et al.: the reported Bland-Altman bias for absolute knee valgus angle lies outside the stated limits of agreement, which is statistically incoherent (by definition, the LoA must encompass the mean bias). This likely indicates a computational or reporting error and precludes reliable interpretation of the agreement analysis.

**Table 3:** Root mean square error comparing 3D markerless motion capture with marker-based systems for jump-landing tasks.

Authors	Time in Which Biomechanical Variables Were Measured	Biomechanical Variable		Root Mean Square Error (Range +/− *SD*)
Aleksic et al.	Entire movement	Ankle angle	Left	5.4 (5.1–5.8)
			Right	6.487 (6.019–6.956)
		Knee angle	Left	6.9 (6.3–7.5)
			Right	7.163 (6.458–7.868)
		Hip angle	Left	8.0 (6.8–9.1)
			Right	8.8 (7.3–10.0)
Drazan et al.	Entire movement	Hip angle (flexion/extension)		1.96
		Knee angle (flexion/extension)		2.68
		Ankle angle (flexion/extension)		3.22
Templin et al.	Entire movement	Hip angle (flexion/extension		6.93 +/− 0.27
		Hip angle (adduction/abduction)		2.52 +/− 1.2
		Hip angle (internal/external rotation)		5.82 +/− 2.45
		Knee angle (flexion/extension		5.55 +/− 2.45
		Knee angle (adduction/abduction)		4.75 +/− 2.15
		Knee angle (internal/external rotation)		5.86 +/− 1.90
		Ankle angle (flexion/extension)		5.77 +/− 1.79
		Ankle angle (adduction/abduction)		9.61 +/− 4.72
Turner et al.	Entire movement	Hip angle (flexion/extension)		5.74 +/− 1.81
		Hip angle (adduction/abduction)		2.39 +/− 0.73
		Hip angle (internal/external rotation)		4.28 +/− 1.28
		Knee angle (flexion/extension)		5.54 +/− 2.18
		Ankle angle (flexion/extension)		6.43 +/− 1.78
		Ankle angle (adduction/abduction)		4.77 +/− 1.34
	Entire movement	Hip angle (flexion/extension)		4.17 +/− 1.29
		Hip angle (adduction/abduction)		3.52 +/− 1.07
		Hip angle (internal/external rotation)		3.73 +/− 1.42
		Knee angle (flexion/extension)		4.98 +/− 1.71
		Ankle angle (flexion/extension)		5.94 +/− 1.91
		Ankle angle (adduction/abduction)		5.19 +/− 1.69
	Entire movement	Hip angle (flexion/extension)		5.02 +/− 2.25
		Hip angle (adduction/abduction)		5.20 +/− 1.44
		Hip angle (internal/external rotation)		5.16 +/− 1.64
		Knee angle (flexion/extension)		6.03 +/− 2.20
		Ankle angle (flexion/extension)		6.87 +/− 2.00
		Ankle angle (adduction/abduction)		5.40 +/− 1.83

**Table 4:** Mean absolute error in comparing 3D markerless motion capture with marker-based systems for jump-landing tasks.

Authors	Time in Which Biomechanical Variables Were Measured	Biomechanical Variable	Mean Absolute Error
Barzyk et al.	Entire movement	Ankle angle (flexion/extension)	4.5 +/− 1.1
		Knee angle (flexion/extension)	2.1 +/− 0.9
		Hip angle (flexion/extension)	2.6 +/− 0.6
Eltoukhy et al.	Entire movement	Hip angle (abduction/adduction)	1.58 +/− 0.89
		Knee angle (flexion/extension)	1.14 +/− 0.40
		Knee angle (adduction/abduction)	1.64 +/− 1.40
	Early deceleration	Hip angle (abduction/adduction)	1.08 +/− 0.82
		Knee angle (flexion/extension)	0.64 +/− 0.82
		Knee angle (adduction/abduction)	0.92 +/− 0.65
Turner et al.	Entire movement	Hip angle (flexion/extension)	4.47 +/− 1.39
		Hip angle (adduction/abduction)	1.91 +/− 0.65
		Hip angle (internal/external rotation)	3.39 +/− 1.10
		Knee angle (flexion/extension)	4.19 +/− 1.61
		Ankle angle (flexion/extension)	5.03 +/− 1.52
		Ankle angle (adduction/abduction)	3.82 +/− 1.19
	Entire movement	Hip angle (flexion/extension)	3.11 +/− 1.01
		Hip angle (adduction/abduction)	2.75 +/− 0.87
		Hip angle (internal/external rotation)	2.93 +/− 1.18
		Knee angle (flexion/extension)	3.49 +/− 1.31
		Ankle angle (flexion/extension)	4.32 +/− 1.52
		Ankle angle (adduction/abduction)	4.07 +/− 1.39
	Entire movement	Hip angle (flexion/extension)	3.75 +/− 1.45
		Hip angle (adduction/abduction)	4.15 +/− 1.17
		Hip angle (internal/external rotation)	4.00 +/− 1.18
		Knee angle (flexion/extension)	4.30 +/− 1.56
		Ankle angle (flexion/extension)	5.41 +/− 1.63
		Ankle angle (adduction/abduction)	4.23 +/− 1.46

**Table 5:** Bland-Altman Plots in comparing 3D markerless motion capture with marker-based systems for jump-landing tasks.

Authors	Time in Which Biomechanical Variables Were Measured	Biomechanical Variable		Bland-Altman Plots		
				Bias (Range)	LoA—Lower (Range)	LoA—Upper (Range)
Aleksic et al.	Entire movement	Ankle angle	Left	−0.49 (−1.45–0.5)	−8.6 (−9.7–−7.6)	7.7 (6.6–8.8)
			Right	−0.67 (−1.96–0.62)	−9.5 (−10.9–−8.1)	8.143 (6.775–9.511)
		Knee angle	Left	2.6 (1.4–3.8)	−6.7 (−7.9–−5.5)	11.9 (10.2–13.6)
			Right	2.01 (0.57–3.44)	−7.3 (−8.7–−5.9)	11.309 (9.440–13.178)
		Hip angle	Left	3.9 (2.5–5.4)	−6.1 (−8.0–−4.288)	14.024 (12–16)
			Right	5.6 (3.8–7.4)	−3.9 (−5.9–−1.8)	15 (12–17)
Asaeda et al.	Entire movement	Absolute knee valgus angle		−19.28	−12.91	25.66
		Knee valgus angle extrusion		0.181	3.165	3.526
Tipton et al.	Peak value	Knee Flexion		10.4	−11.7	26.8
		Knee valgus		5.31	−8	13.9
	Peak value	Knee Flexion		7.96	−17.7	21.3
		Knee valgus		4.69	−6.3	12.6
	Peak value	Knee Flexion		7.39	−17.8	19.7
		Knee valgus		4.22	−5.9	11.6

**Table 6:** Intraclass correlation coefficient (2,1) in comparing 3D markerless motion capture with marker-based systems for jump-landing tasks.

Authors	Time in Which Biomechanical Variables Were Measured	Biomechanical Variable		ICC(2,1) (95% Confidence Interval)	*p*-Value
Eltoukhy et al.	Entire movement	Hip angle (abduction/adduction)		0.873 (0.476–0.971)	Data not given
		Knee angle (flexion/extension)		0.973 (0.886–0.994)	Data not given
		Knee angle (adduction/abduction)		0.767 (0.102–0.946)	Data not given
	Early deceleration	Hip angle (abduction/adduction)		0.643 (−0.697–0.921)	Data not given
		Knee angle (flexion/extension)		0.989 (0.0.923–0.998)	Data not given
		Knee angle (adduction/abduction)		0.831 (0.328–0.961	Data not given
Gray et al.	Initial contact	Knee-ankle separation ratio (KASR)		0.84	Data not given
	Peak flexion	Knee-ankle separation ratio (KASR)		0.95	Data not given
Mauntel et al.	Initial contact (IC)	Hip flexion	Left	0.5	0.1
			Right	0.63	0.03
		Hip angle (adduction/abduction)	Left	0.61	<0.001
			Right	0.47	0.01
		Knee flexion	Left	0.96	<0.001
			Right	0.95	<0.001
		Knee valgus	Left	−0.19	0.64
			Right	0.21	0.31
	Maximum value (during the landing phase)	Hip flexion	Left	0.86	<0.001
			Right	0.86	<0.001
		Hip adduction	Left	0.668	0.002
			Right	0.55	0.05
		Hip abduction	Left	0.63	0.001
			Right	0.6	0.02
		Knee flexion	Left	0.85	<0.001
			Right	0.75	0.003
		Knee valgus	Left	0.83	<0.001
			Right	0.92	<0.001
	Joint displacement (from IC to Max during landing)	Hip flexion	Left	0.92	<0.001
			Right	0.91	<0.001
		Hip adduction	Left	−0.4	0.55
			Right	0.02	0.49
		Hip abduction	Left	0.67	0.01
			Right	0.17	0.3
		Knee flexion	Left	0.85	<0.001
			Right	0.76	0.003
		Knee valgus	Left	−1.52	0.97
			Right	0.8	0.001
Stone et al.	Entire movement	Knee Valgus motion	Left	0.81 (0.72–0.87)	Data not given
			Right	0.85 (0.77–0.91)	Data not given
	Initial contact	Coronal plane knee angle (varus/valgus alignment)	Left	Approx. 0.89 (0.74–0.95)	Data not given
			Right	Approx. 0.89 (0.81–0.94)	Data not given
	Peak knee flexion	Coronal plane knee angle (varus/valgus alignment)	Left	Approx. 0.89 (0.77–0.95)	Data not given
			Right	Approx. 0.89 (0.85–0.93)	Data not given
	Peak knee flexion	Knee-to-ankle separation ratio		Approx. 0.89 (0.84–0.93)	Data not given
Tipton et al.	Peak value	Knee Flexion		0.654 (0.435–0.8)	Data not given
		Knee valgus		0.589 (0.231–0.785)	Data not given
	Peak value	Knee Flexion		0.759 (0.587–0.866)	Data not given
		Knee valgus		0.717 (0.393–0.862)	Data not given
	Peak value	Knee Flexion		0.594 (0.166–0.801)	Data not given
		Knee valgus		0.553 (0.253–0.747)	Data not given

**Table 7:** Pearson’s correlation coefficient in comparing 3D markerless motion capture with marker-based systems for jump-landing tasks (ms = milliseconds).

Authors	Time in Which Biomechanical Variables Were Measured	Biomechanical Variable		Pearson’s Correlation Coefficient	
				*r* (Range +/− *SD*)	*p*-Value
Aleksic et al.	Entire movement	Ankle angle	Left	0.984 (0.982–0.986)	Data not given
			Right	0.981 (0.978–0.986)	Data not given
		Knee angle	Left	0.994 (0.993–0.995)	Data not given
			Right	0.994 (0.993–0.995)	Data not given
		Hip angle	Left	0.997 (0.996–0.007)	Data not given
			Right	0.996 (0.995–0.007)	Data not given
Asaeda et al.	Initial contact	Absolute knee valgus angle		0.016	0.954
	10 ms after initial contact	Absolute knee valgus angle		0.017	0.951
		Knee valgus angle extrusion (from initial contact)		0.554	0.032
	20 ms after initial contact	Absolute knee valgus angle		0.008	0.976
		Knee valgus angle extrusion (from initial contact)		0.604	0.017
	30 ms after initial contact	Absolute knee valgus angle		0.138	0.623
		Knee valgus angle extrusion (from initial contact)		0.697	0.004
	40 ms after initial contact	Absolute knee valgus angle		0.181	0.517
		Knee valgus angle extrusion (from initial contact)		0.757	0.001
	50 ms after initial contact	Absolute knee valgus angle		0.145	0.606
		Knee valgus angle extrusion (from initial contact)		0.673	0.006
	60 ms after initial contact	Absolute knee valgus angle		0.214	0.443
		Knee valgus angle extrusion (from initial contact)		0.67	0.006
	70 ms after initial contact	Absolute knee valgus angle		0.368	0.178
		Knee valgus angle extrusion (from initial contact)		0.664	0.007
	80 ms after initial contact	Absolute knee valgus angle		0.494	0.061
		Knee valgus angle extrusion (from initial contact)		0.673	0.006
	90 ms after initial contact	Absolute knee valgus angle		0.59	0.021
		Knee valgus angle extrusion (from initial contact)		0.714	0.003
	100 ms after initial contact	Absolute knee valgus angle		0.475	0.074
		Knee valgus angle extrusion (from initial contact)		0.629	0.012
Barzyk et al.	Entire movement	Ankle angle (flexion/extension)		0.87 +/− 0.08	Data not given
		Knee angle (flexion/extension)		0.99 +/− 0.01	Data not given
		Hip angle (flexion/extension)		0.96 +/− 0.04	Data not given
Eltoukhy et al.	Entire movement	Hip angle (abduction/adduction)		0.854 (0.269–0.911)	<0.05
		Knee angle (flexion/extension)		0.961 (0.591–0.999)	<0.05
		Knee angle (adduction/abduction)		0.847 (0.181–0.641)	<0.05
	Early deceleration	Hip angle (abduction/adduction)		0.471 (−0.242–0.961)	>0.05
		Knee angle (flexion/extension)		0.985 (0.825–1.131)	<0.05
		Knee angle (adduction/abduction)		0.732 (0.12–1.301)	<0.05
Templin et al.	Entire movement	Hip angle (flexion/extension		0.98 +/− 0.02	Data not given
		Hip angle (adduction/abduction)		0.69 +/− 0.34	Data not given
		Hip angle (internal/external rotation)		0.67 +/− 0.29	Data not given
		Knee angle (flexion/extension		0.99 +/− 0.01	Data not given
		Knee angle (adduction/abduction)		0.45 +/− 0.43	Data not given
		Knee angle (internal/external rotation)		0.59 +/− 0.37	Data not given
		Ankle angle (flexion/extension)		0.97 +/− 0.01	Data not given
		Ankle angle (adduction/abduction)		0.06 +/− 0.48	Data not given

**Table 8:** Coefficient of multiple correlation in comparing 3D markerless motion capture with marker-based systems for jump-landing tasks.

Authors	Time in Which Biomechanical Variables Were Measured	Biomechanical Variable	Coefficient of Multiple Correlation (+/− *SD*)
Drazan et al.	Entire movement	Hip angle (flexion/extension)	0.991
		Knee angle (flexion/extension)	0.995
		Ankle angle (flexion/extension)	0.995
Turner et al.	Entire movement	Hip angle (flexion/extension)	0.98 +/− 0.02
		Hip angle (adduction/abduction)	0.65 +/− 0.05
		Hip angle (internal/external rotation)	0.60 +/− 0.60
		Knee angle (flexion/extension)	0.98 +/− 0.01
		Ankle angle (flexion/extension)	0.93 +/− 0.06
		Ankle angle (adduction/abduction)	0.48 +/− 0.19
	Entire movement	Hip angle (flexion/extension)	0.94 +/− 0.06
		Hip angle (adduction/abduction)	0.78 +/− 0.13
		Hip angle (internal/external rotation)	0.51 +/− 0.23
		Knee angle (flexion/extension)	0.94 +/− 0.07
		Ankle angle (flexion/extension)	0.84 +/− 0.14
		Ankle angle (adduction/abduction)	0.54 +/− 0.21
	Entire movement	Hip angle (flexion/extension)	0.94 +/− 0.08
		Hip angle (adduction/abduction)	0.75 +/− 0.16
		Hip angle (internal/external rotation)	0.56 +/− 0.20
		Knee angle (flexion/extension)	0.97 +/− 0.02
		Ankle angle (flexion/extension)	0.85 +/− 0.12
		Ankle angle (adduction/abduction)	0.47 +/− 0.18

## Data Availability

No new data were created or analyzed in this study. Data sharing is not applicable to this article.
